# Secreted Interferon-Inducible Factors Restrict Hepatitis B and C Virus Entry In Vitro

**DOI:** 10.1155/2017/4828936

**Published:** 2017-03-06

**Authors:** Yuchen Xia, Xiaoming Cheng, Christoph K. Blossey, Karin Wisskirchen, Knud Esser, Ulrike Protzer

**Affiliations:** ^1^Institute of Virology, Technische Universität München/Helmholtz Zentrum München, 81675 Munich, Germany; ^2^German Center for Infection Research (DZIF), Partner Site Munich, Munich, Germany

## Abstract

Interferon-*α* (IFN-*α*) has been used for more than 20 years as the first-line therapy for hepatitis B virus (HBV) and hepatitis C virus (HCV) infection, because it has a number of antiviral effects. In this study, we describe a novel mode of its antiviral action. We demonstrate that the supernatant from IFN-*α*-treated cultured cells restricted HBV and HCV infection by inhibiting viral entry into hepatoma cells. The factors contained in the supernatant competed with the virus for binding to heparan glycosaminoglycans—the nonspecific attachment step shared by HBV and HCV. Secreted factors of high molecular mass that bind to heparin columns elicited the antiviral effect. In conclusion, IFN-*α* is able to induce soluble factors that can bind to heparan glycosaminoglycans thus leading to the inhibition of viral binding.

## 1. Introduction

Interferon (IFN) was first discovered in 1957, based on the observation that cells challenged with heat-inactivated influenza virus secreted a macromolecule that was able to “interfere” with several viruses, such as the infectious influenza virus or vaccinia virus [1]. This study and follow-up researches led to the development of IFN as a therapeutic tool to treat a number of infectious diseases, in particular chronic hepatitis B and hepatitis C. IFN elicits antiviral actions by inducing a wide array of IFN-stimulated genes (ISGs). By interacting with their specific receptors, IFN activates signal transducer and activator of transcription (STAT) complexes. Phosphorylated STAT then activates the classical Janus kinase-STAT signaling pathway and initiates the transcription of different ISGs [2].

The mechanisms by which these ISGs participate in the IFN-mediated response to hepatitis B virus (HBV) or hepatitis C virus (HCV) are not fully understood. Previous studies demonstrated that IFN inhibits HBV replication at multiple steps of its life cycle, by deaminating and degrading the viral transcription template covalently closed circular DNA (cccDNA) through apolipoprotein B mRNA editing enzyme catalytic subunit 3A [3–5], silencing cccDNA through epigenetic regulation by the STAT complex [6, 7], downregulating viral mRNA stability through antiviral zinc finger proteins [8, 9], inhibiting viral pregenomic RNA encapsidation via myxoma resistance protein 1 [10, 11], and reducing virion secretion by Tetherin [12]. IFN also promotes viral nucleocapsid degradation [13].

HCV and host ISGs show a much more complex interaction. For example, cellular pattern recognition receptors detect molecular patterns of HCV [14, 15], thereby forming a positive feedback loop to amplify IFN signaling. Many ISGs are reported to inhibit HCV RNA replication or viral protein translation, either directly or indirectly, including RNA-specific adenosine deaminase [16], viperin [17], and 2′-5′-oligoadenylate synthetase [18]. IFN also induces transmembrane protein 1 (IFITM1) which was reported to inhibit HCV entry [19].

Virus entry can be a multistep process, in which the virus first attaches to cell type unspecific molecules, then binds to its specific receptor, and enters the cell. Heparan sulfate is present on the surface and in the extracellular matrix of all mammalian cells and serves as an attachment factor or anchor for a number of enveloped viruses such as herpes simplex virus [20], respiratory syncytial virus [21], human immunodeficiency virus [22], cytomegalovirus [23], Dengue virus [24], HBV [25], and HCV [26], as well as nonenveloped viruses such as human papillomavirus [27] and foot-and-mouth disease virus [28].

Although the influence of ISG products on the replication of HBV or HCV has been studied extensively, little is known about the influence of IFN treatment on early steps of the virus life cycle. Here, we investigated whether IFN-*α* was able to induce soluble factors that would have extracellular antiviral activity. Our study reveals a novel antiviral mechanism of IFN-*α*. Upon IFN-*α* treatment factors are secreted that bind to heparan glycosaminoglycans—the attachment receptor of many viruses including HBV and HCV—thus leading to the inhibition of virus attachment and blocking infection.

## 2. Materials and Methods

### 2.1. Cell Cultures

HepaRG cells were cultured in Williams E medium (Gibco, Carlsbad, USA) supplemented with 10% fetal calf serum FetalClone II (HyClone, Little Chalfont, United Kingdom), 20 mM L-glutamine (Gibco, Carlsbad, USA), 50 U/mL penicillin/streptomycin (Gibco, Carlsbad, USA), 80 *μ*g/mL gentamicin (Ratiopharm, Ulm, Germany), 0.023 IE/mL human insulin (Sanofi-Aventis, Paris, France), and 4.7 *μ*g/mL hydrocortisone (Pfizer, Carlisle, USA) as described [29]. The cell cultures were maintained in a 5% CO2 atmosphere at 37°C. For infection, cells were maintained for 2 weeks in standard medium and then differentiated for 2 more weeks in medium supplemented with 1.8% DMSO (Sigma, Munich, Germany).

### 2.2. HBV Production

HBV was concentrated from the supernatant of HepG2.2.15 cells using centrifugal filter devices (Centricon Plus-70, Biomax 100.000, Millipore Corp., Bedford, MA) and quantified by HBV-DNA qPCR. Immediately after collection, the virus stock was divided into aliquots and stored at −80°C until use.

### 2.3. HBV Infection

The inoculation of differentiated HepaRG cells was performed with a multiplicity of infection of 200 (genome copies per cell) in differentiation medium containing 5% PEG 8000 (Sigma, Munich, Germany) for 16 h at 37°C. At the end of the incubation period, cells were washed three times with PBS and cultured in differentiation medium.

### 2.4. Analysis of HBV Replication

HBsAg was measured using the AXSYM system (Abbott, Chicago, USA), and HBeAg was measured by the BEP III system (Siemens, Munich, Germany). Total cellular DNA or DNA from cell culture supernatant were extracted from infected cells using NucleoSpin Tissue Kit (Macherey-Nagel, Düren, Germany). Real-time quantitative PCRs (qPCRs) were performed using the LightCycler™ system (Roche, Manheim, Germany) and HBV-DNA and cccDNA were detected using specific PCR primers (Table 1). HBV-DNA from cell culture supernatant was quantified relative to an external plasmid standard. Intracellular HBV-DNA and cccDNA are expressed as normalized ratio to the genomic single copy gene of the prion protein (PRNP).

### 2.5. Western Blot

Lysates from HepaRG cells were obtained by adding 200 *μ*L “M-PER Mammalian Protein Extraction Reagent” (Thermo Scientific, Schwerte, Germany) onto cells per well and incubated at 37°C for five minutes. 50 *μ*L “LDS sample buffer Nonreducing” was added and samples were shaken for five minutes at 800 rpm, 99°C. Proteins were separated on 7.5% sodium dodecyl sulfate-polyacrylamide gel electrophoresis. Then, proteins were blotted onto a PVDF membrane. The membrane was blocked with 5% milk for one hour at room temperature followed by overnight incubation with polyclonal rabbit anti-HBV core antibody (gift from Heinz Schaller) at 4°C.

### 2.6. Virus Heparin Attachment Assay

96-well plates were coated with 100 *μ*L of a 25 *μ*g/mL heparin solution per well and incubated at 4°C overnight. The wells were washed 3 times with PBS. Then 100 *μ*L of samples was added and the plate was incubated overnight at 4°C. The samples were aspirated and the plate was washed 3 times with PBS. 300 *μ*L of blocking buffer (1% BSA solution in PBS-T) was added to each well and incubated for 2 h at 37°C. After that, the plate was washed 3 times with PBS-T.

100 *μ*L of blocking buffer, 1 *μ*L of virus stock solution, and 50 *μ*L Murex Conjugate solution (from Murex HBsAg Version 3 Kit, Saluggia, Italy) were added per well. The plate was incubated at 37°C for 1 h followed by 4 times with PBS-T washing. Then, 100 *μ*L of substrate solution was added per well. After 1 h incubation at 37°C, 50 *μ*L stop solution (1 M H_2_SO_4_ in ddH2O) was added per well. Light absorption was measured at 450 nm, with 670 nm reference wavelengths with an Infinite 200 (Tecan, Männedorf, Switzerland).

### 2.7. Enzymes Treatment Assay

Samples were treated with 50 U/mL N-glycosidase F beads (EDM Millipore, Bedford, MA) overnight at 37°C, or 0.0005% Trypsin (Gibco, Carlsbad, USA) for 1 hour at 37°C, or 0.1 *μ*g/mL proteinase K for 1 hour at 37°C. After incubation, 5 mM protease inhibitor Pefabloc (Sigma, Munich, Germany) was added to the samples. Finally, the samples were purified with Vivaspin MWCO 3000 SpinColumn (Gelifesciences, Freiburg, Germany).

### 2.8. Preparation of Luciferase Reporter HCV Stocks

Huh7.5 cells were electroporated with reporter constructs pFK-Luc-Jc1 as described previously [30]. Culture supernatant of transfected cells was harvested and filtered through 0.45 *μ*m Stericup® Filter Units (SCHVU11RE, Millipore, Germany) and precipitated with 8% PEG8000 at 4°C overnight. Virus was pelleted by centrifugation at 5000 ×g for 2.5 h at 4°C, resuspended in DMEM, and stored at −80°C before use. Each preparation was titrated for its infectivity by limiting dilution assay on Huh7.5 cells and 50% and the tissue culture infective dose (TCID_50_) was calculated based on the method described [31].

### 2.9. HCV Infection and Luciferase Assay

Huh7.5 cells were seeded in a 96-well plate (1 × 10^4^/well) for 24 h prior to HCV inoculation. 0.1 MOI/cell of HCV luciferase virus were used to infect the cells for 72 h. 10 *μ*L of prepared “elution” and “concentrate” was present in the 200 *μ*L culture medium during the whole process of HCV infection. Luciferase assays were performed using the assay kit (E1500, Promega, USA) following manufacturer's instructions. Luminescence was measured in programmed inject-then-read mode (Infinite F200, Männedorf, Switzerland). All luciferase assays were performed in triplicate.

### 2.10. Statistical Analysis

Student's unpaired two-tailed *t*-tests were performed with GraphPad Prism 5.0a (GraphPad Software, La Jolla, CA, USA). Data are means ± s.d. Two-sided *P* values <0.05 were considered significant. ^*∗*^*P* < 0.05, ^*∗∗*^*P* < 0.01, and ^*∗∗∗*^*P* < 0.001.

## 3. Results

### 3.1. Pretreatment with IFN-*α* Inhibits HBV Replication

**Figure 1:**
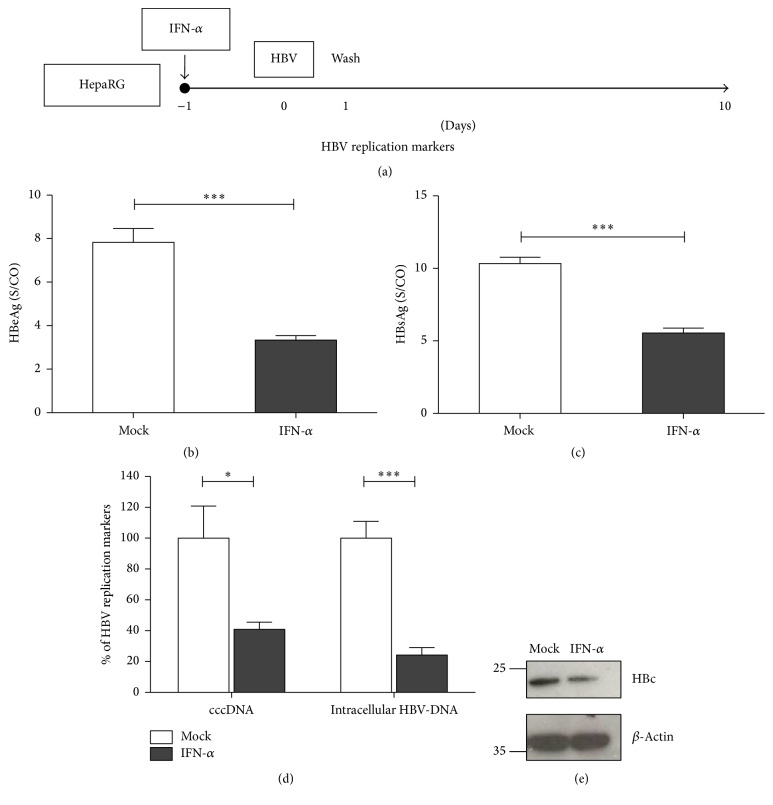
IFN-*α* pretreatment inhibits HBV infection. Differentiated HepaRG cells were treated with 1000 IU/mL of IFN-*α* for 1 day (IFN-a(−1)) and then infected with HBV (MOI = 200) (a). HBeAg from cell culture supernatant was measured by ELISA at day 10 (b). HBsAg was measured at day 7 and day 10 (c). HBV cccDNA and intracellular DNA were evaluated by qPCR (d). HBV core was detected by Western blot (e). Data are means ± s.d. ^*∗*^*P* < 0.05, ^*∗∗*^*P* < 0.01, and ^*∗∗∗*^*P* < 0.001 by Student's unpaired two-tailed *t*-test.

We first sought to investigate whether pretreatment of HepaRG cells with IFN-a before infection with HBV would have an effect on establishment of HBV infection (Figure 1(a)). IFN-*α* pretreatment resulted in a decline of HBeAg and HBsAg at day 10 upon HBV infection (Figures 1(b) and 1(c)). Concomitantly, intracellular HBV replication markers at day 10 were analyzed, and qPCR analysis revealed more than 50% reduction of cccDNA and intracellular HBV-DNA after IFN-*α* pretreatment (Figure 1(d)). Western blot analysis showed the decline of intracellular HBV core protein production (Figure 1(e)). These results suggested that the pretreatment of cells with IFN-*α* for 24 hours is sufficient to induce an antiviral effect and the IFN-induced antiviral factors sustained this activity during infection. These results also implied that IFN-*α* induces an antiviral program preventing HBV early infection.

### 3.2. Interferon-Inducible Secreted Factors Restrict Early Steps of HBV Infection

**Figure 2:**
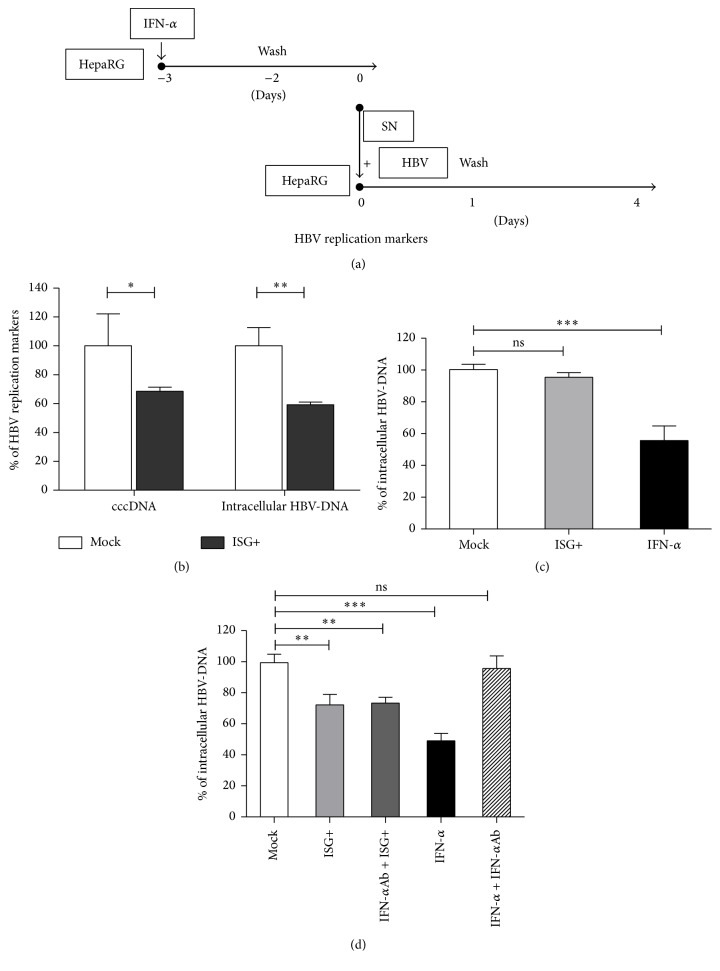
Secreted ISGs restrict early steps of HBV infection. (a) Differentiated HepaRG cells were treated with 1000 IU/mL of IFN-*α*. One day later, the medium was removed. Cells were washed three times with PBS and refilled with new medium. The resulting medium containing interferon induced factors (ISG+) was collected 48 hours later and then mixed with HBV and PEG. The mixture then transferred onto fresh differentiated HepaRG cell for HBV infection. (b) HBV cccDNA and intracellular DNA were evaluated by qPCR 4 days after infection. (c) Differentiated HepaRG cells were infected with HBV (MOI = 200) and 7 days later different treatments were applied as indicated. Intracellular HBV-DNA was measured 4 days after treatment. (d) Differentiated HepaRG cells were incubated with indicated treatment for 24 hours and then infected with HBV (MOI = 200). Intracellular HBV-DNA was measured 4 days after infection. Data are means ± s.d. ^*∗*^*P* < 0.05, ^*∗∗*^*P* < 0.01, and ^*∗∗∗*^*P* < 0.001 by Student's unpaired two-tailed *t*-test.

In order to determine whether the IFN-*α* induced antiviral program would prevent initiation of HBV replication or inhibit attachment or entry into the host cell a conditioned medium was prepared from IFN-*α* treated cells (Figure 2(a)). The medium containing the ISG products (ISG+) was collected and added together with HBV to differentiated HepaRG cells. Compared with untreated cells, ISG+ medium treated cells showed decreased cccDNA and intracellular HBV-DNA demonstrating the inhibitory effect of ISG+ medium (Figure 2(b)). The ISG+ medium did not have an effect when it was applied to cells that had already been infected (Figure 2(c)).

To exclude that the antiviral action elicited by ISG+ medium was due to residual IFN-*α*, neutralizing antibodies (IFN-*α*-Ab) were applied. When either IFN-*α* or ISG+ medium was added concomitantly with the HBV inoculum, HBV infection was inhibited, but only the effect of IFN-*α* but not that of ISG+ medium was neutralized by IFN-*α*Ab (Figure 2(d)). This proved that the antiviral activity of ISG+ medium was not elicited by residual IFN-*α*.

Taken together, the experiments show that ISG+ medium decreased HBV infection in cells treated before or during HBV infection, whereas no effect was observed if cells were treated following HBV infection. Thus we concluded that IFN-*α* treatment induces hepatoma cells to secrete soluble factors, and those soluble factors inhibit HBV infection by targeting an early step of HBV infection, most likely virus entry.

### 3.3. Interferon Induced Factors Interrupt HBV Binding

**Figure 3:**
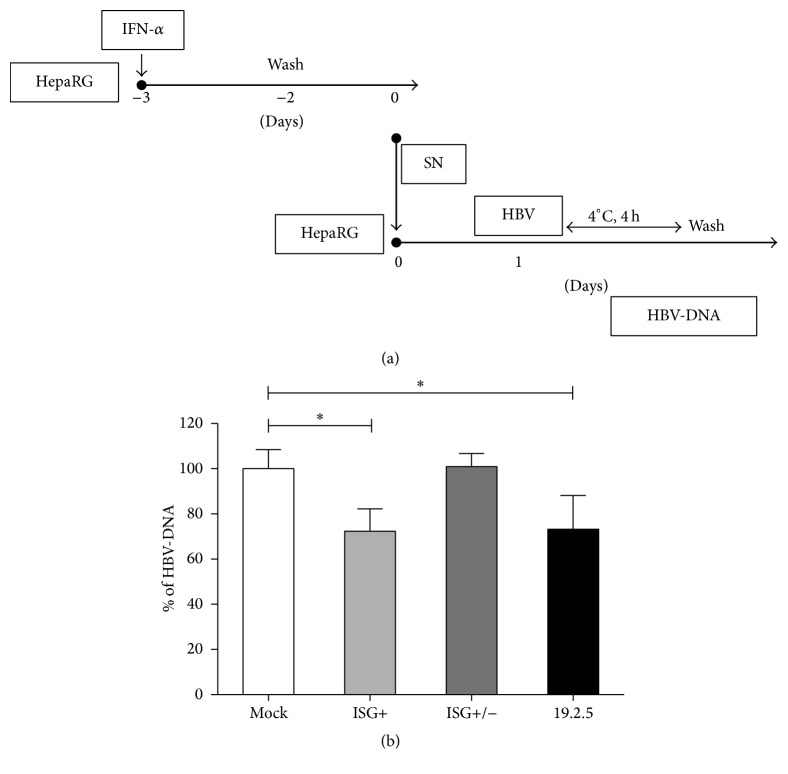
Secreted ISGs interrupt HBV binding. (a) Differentiated HepaRG cells were treated with 1000 IU/mL of IFN-*α*. One day later, the medium was removed. Cells were washed three times with PBS and refilled with new medium. The resulting medium containing interferon induced factors (ISG+) was collected 48 hours later. Part of the medium was heated at 99°C for 10 minutes to inactivate proteins (ISG+/−). The medium was incubated with fresh differentiated HepaRG cells for 24 hours followed by 4 hours of HBV incubation at 4°C. Peptide 19-2.5 was used as a positive control. (b) Cells were lysed and HBV-DNA were evaluated by qPCR. Data are means ± s.d. ^*∗*^*P* < 0.05, ^*∗∗*^*P* < 0.01, and ^*∗∗∗*^*P* < 0.001 by Student's unpaired two-tailed *t*-test.

To further characterize the effect of interferon induced factors, we performed a virus attachment assay (Figure 3(a)). Differentiated HepaRG cells were incubated with mock (untreated differentiated HepaRG cell culture supernatant), ISG+ medium, heat-inactivated ISG+ medium (ISG+ inactivated), or the synthetic antilipopolysaccharide peptide Pep19-2.5, which served as a positive control as it has been shown to inhibit the binding step of many enveloped viruses including HBV [32]. Cells were then inoculated with virus particles for 4 hours at 4°C, because low temperature prohibits HBV entry into the host cell. Hereby, we found that interferon induced soluble factors could inhibit HBV binding as efficiently as Pep19-2.5, and heat inactivation led to attenuation of the antiviral effect (Figure 3(b)).

These results demonstrate that HBV binding to its host cell was impeded by treatment with ISG+ medium containing interferon induced soluble factors. We therefore speculated that the induced antiviral proteins associated with the virus itself or with one of the essential viral attachment factors or receptors.

### 3.4. Interferon Induced Factors Interrupt HBV Binding to Heparin Sulfate

**Figure 4:**
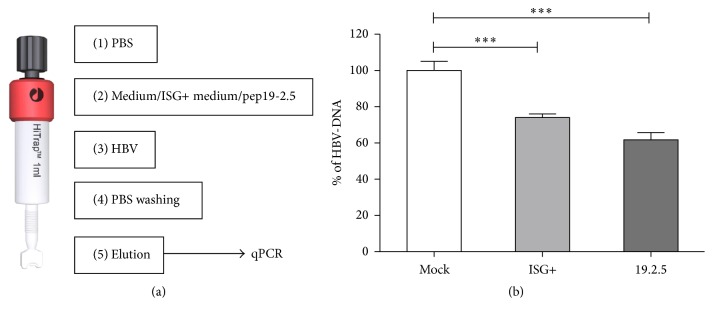
IFN-*α* induced products compete with HBV for binding to heparin column. (a) Heparin columns were washed with PBS and then applied with differentiated HepaRG cell culture medium (mock) or IFN-*α* treated differentiated HepaRG cell culture medium (ISG+) or the entry inhibitor Pep19-2.5. Then columns were applied with PBS containing HBV. After washing, HBV was eluted by elution buffer. (b) HBV amount from different elutions were analyzed by HBV-DNA qPCR.

Experimental evidence has been presented that HBV initiates infection of hepatocytes by binding to heparan sulfate proteoglycans [25]. We therefore analyzed whether IFN-*α* induced factors can interrupt the interaction between HBV and heparin, which is a close homologue of liver heparin sulfate [33]. We applied cell culture supernatant of untreated, differentiated HepaRG cells, ISG+ medium, or Pep19-2.5 on heparin columns and analyzed the residual binding capacity of HBV (Figure 4(a)). HBV virions efficiently bound to heparin-Sepharose under physiological salt conditions (mock) and could be eluted using high salt concentration (Figure 4(b)). HBV particle binding to heparin was reduced by 30% when particles were loaded after ISG+ medium. Hereby ISG+ medium inhibited HBV binding to heparin column almost as efficiently as Pep19-2.5 that has been described previously [32]. These results demonstrate that ISG+ medium competes with HBV for binding to heparin.

**Figure 5:**
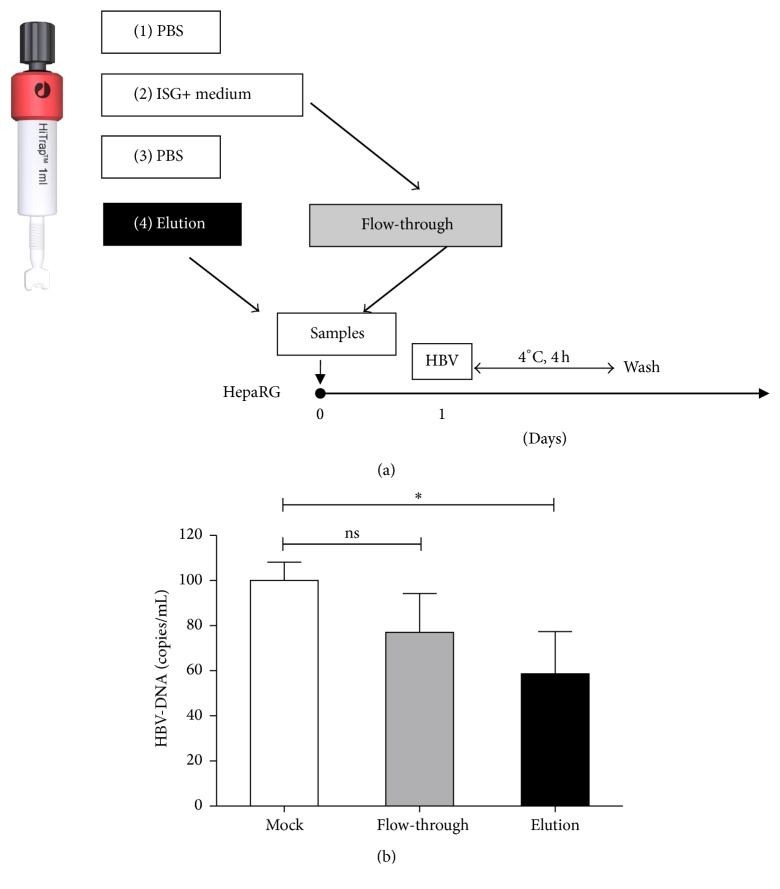
IFN-*α* induced products inhibit HBV binding to the cells. (a) Heparin columns were washed with PBS and then applied with IFN-*α* treated differentiated HepaRG cell culture medium. Then columns were washed with PBS. Differentiated HepaRG cells were incubated with elution or flow-through for 24 hours followed by 4 hours of HBV incubation at 4°C. (b) Cells were lysed and cellular HBV-DNA were evaluated by qPCR. Data are means ± s.d. ^*∗*^*P* < 0.05, ^*∗∗*^*P* < 0.01, and ^*∗∗∗*^*P* < 0.001 by Student's unpaired two-tailed *t*-test.

To further investigate our hypothesis that soluble factors in the ISG+ medium competitively bind to heparin in cultured cells, ISG+ medium was applied to the heparin column, and the flow-through (low heparin affinity part) and the elution (high heparin affinity part) were collected and applied to differentiated HepaRG cells (Figure 5(a)). The elution fraction from the heparin column containing high heparin affinity factors led to a reduction of HBV binding to HepaRG cells while the flow-through fraction showed no significant inhibition (Figure 5(b)). In conclusion, these results show that interferon induced factors inhibit initial binding of HBV to the cell surface by direct interaction with heparan sulfate.

### 3.5. Characterization of IFN-*α* Induced Binding Inhibitors

**Figure 6:**
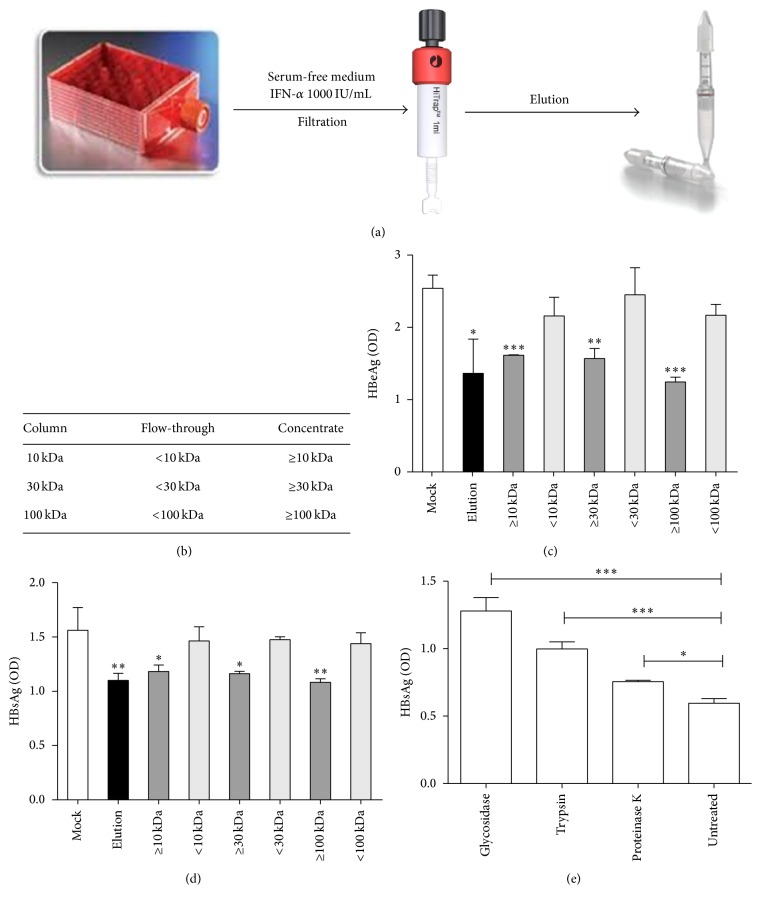
Size exclusion of IFN-*α* induced binding inhibitors. (a) HepaRG cells were cultivated in hyperflask. After differentiation, cells were stimulated with 1000 IU/mL IFN-*α* for one day and then fed fresh serum-free medium. The heparin binding fraction was purified from heparin columns and protein concentration columns with different cutoffs were used to further separate the fractions by protein size. (b) Protein size of each fraction. (c) Differentiated HepaRG cells were incubated with indicated samples for 24 hours and then infected with HBV. HBeAg was evaluated by ELISA 4 days after infection. (d) Highly purified HBV SVPs from chronic HBV carriers were added to heparin-coated (25 *μ*g/mL) 96-well plate and treated with different fractions. Plates were incubated for 2 h at 37°C, and heparin-bound SVPs were detected using an HBsAg ELISA kit. (e) Differentiated HepaRG cells were incubated with elution treated by glycosidase, or trypsin, or proteinase K for 24 hours and then infected with HBV. HBsAg was evaluated by ELISA 4 days after infection.

In order to characterize the active factors in ISG+ medium in more detail, we determined their size using size exclusion chromatography. The heparin binding fraction was purified and applied to size exclusion columns with a cut-off of 10 kDa, 30 kDa, and 100 kDa, respectively, to further concentrate and separate the fractions by protein size (Figure 6(a)). Both flow-through and concentrate from each column were collected, and six different fractions containing a different size range of proteins (Figure 6(b)) were incubated on differentiated HepaRG cells before HBV inoculation. Elution from heparin columns inhibited HBV replication as expected. Among different fractions, all three concentrates containing proteins bigger than 100 kDa showed antiviral effects while all three flow-through fractions containing proteins smaller than 100 kDa did not reduce infection (Figure 6(c)).

To confirm binding specificity of the IFN-*α* induced factors to heparin, we coated a plate with heparin and added the elution from heparin columns or different fractions from protein concentration columns. We then determined the amount of HBV that could still bind to the heparin plate (Figure 6(d)). The elution fraction or the respective concentrates from all size exclusion chromatography columns competed with HBV for binding to heparin. The three flow-through samples did not show any inhibition of HBV binding. Taken together, this suggested that the interferon induced secreted factors that inhibit binding of HBV to heparin are larger than 100 kDa.

To further characterize the IFN-*α* induced binding inhibitors, we treated heparin column purified ISG+ medium (elution) with glycosidase, trypsin, or proteinase K (Figure 6(e)). Interestingly, all three enzyme treatments enhanced HBV replication, which indicated that the antiviral factors present in the ISG+ medium are glycoproteins. The strong inhibitory effect of glycosidase on the factors suggested that carbohydrate groups of the glycoproteins might play an important role in the interaction with heparan sulfate.

### 3.6. Interferon Induced Secreted Factors Restrict HCV Infection

**Figure 7:**
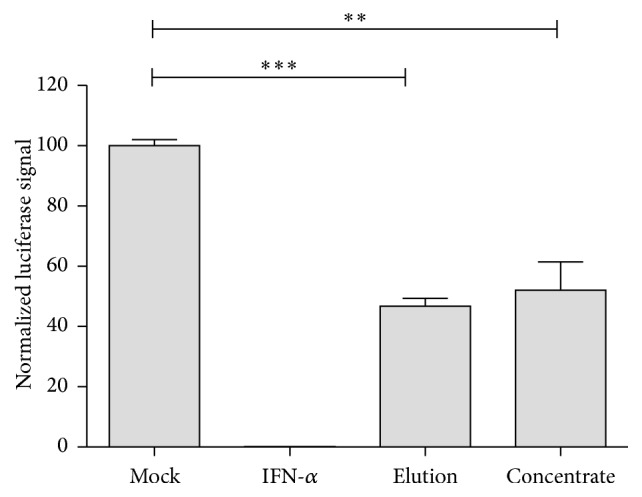
IFN-*α* induced factors inhibit HCV replication. Huh7.5 cells were seeded in 96-well plates in triplicate 24 h prior to virus infection. HCV luciferase reporter virus was used to infect cells at an MOI of 0.1 TCID50/cell. 1000 IU/mL of IFN-*α*, 10 *μ*L of prepared “elution” or “concentrate” was added to the culture medium (total volume = 200 *μ*L) from the beginning of inoculation until the cells were lysed 72 h later after HCV infection. Luciferase activity was determined and one of two independent experiments is displayed as mean ± SD.

HCV is an alternative hepatotropic virus that uses heparan sulfate as a primary docking site for infection [26]. To investigate whether IFN-induced secreted factors were able to also inhibit HCV infection, IFN-*α* and heparin column elution or concentrate from 100 kDa protein concentration columns were added during infection with HCV luciferase virus (Figure 7). Importantly, both elution fractions from heparin columns and concentrates from size exclusion columns also restricted HCV infection by 50% (Figure 7). This result confirms that IFN inducible secreted factors block HCV binding to heparin sulfate proteoglycans and thus are able to block this common step of the two hepatotropic viruses, HBV and HCV.

## 4. Discussion

In the present study, we describe a novel antiviral mechanism of IFN-*α* that targets the HBV and HCV binding step. The supernatant of IFN-*α* treated cell cultures restricts HBV and HCV entry and infection. The inhibition is contributed by one or more secreted interferon induced proteins, which bind to heparin columns. This result indicates that the proteins in the IFN-*α* treated cell culture supernatant can bind to heparan glycosaminoglycans—the unspecific attachment receptor of many viruses.

Although ISG+ medium was prepared from IFN-*α* treated cells, the antiviral activity of ISG+ medium was unlikely due to residual amount of IFN-*α* present in the conditioned medium or de novo synthesized IFN-*α*/*β* in ISG+ medium treated cells. First, the residual IFN-*α*, if there was any, would be fast endocytosed and rapidly degraded by ISG producing cells [34]. Second, IFN-*α* treatment does not induce IFN-*α*/*β* production [35].

Initial interactions between an enveloped virus and its host cell are normally mediated through its membrane glycoproteins by binding to glycolipids and/or glycoprotein attachment factors, such as heparan sulfate proteoglycans, on the target cell surface [36]. For HBV, this first encounter is initiated via interaction of the “a” determinant(s) with heparan sulfate proteoglycans, resulting in the large envelope protein being able to bind to its specific receptor sodium-taurocholate cotransporting polypeptide to allow viral entry [25, 37]. Our enzyme digestion experiment suggested that IFN-*α* induced glycoproteins could compete with viral membrane proteins for binding to heparin sulfate proteoglycans thus blocking the whole entry process of virus infection.

IFN-*α* induced antiviral response is known to be multifunctional for a long time; however, its effects on the virus binding or entry steps are not well studied [38]. Until recently, the interferon-inducible transmembrane (IFITM) protein family has been shown to block early stages of viral infection [39, 40]. IFITM was originally identified through RNAi genetic screening and was shown to inhibit infections of vesicular stomatitis virus (VSV), influenza A virus, West Nile virus, and Dengue virus. Later IFITM proteins were found to potently restrict entry and infections by a number of highly pathogenic viruses, including human immunodeficiency virus (HIV), filoviruses, HCV, and SARS coronavirus [17, 19, 41–44]. Recently, researchers identified cholesterol-25-hydroxylase (CH25H) as a broad antiviral ISG [45]. CH25H converts cholesterol into a soluble antiviral factor, 25-hydroxycholesterol (25HC). 25HC treatment in cultured cells inhibited growth of a broad group of enveloped viruses including VSV, HIV, and herpes simplex virus. Interestingly, it also blocks HBV entry [46]. Since the molecular weight of IFITM or CH25H is less than 100 kDa, other factors that may contribute to the antiviral effect we observed.

The specific inhibition of virus entry is an attractive therapeutic target not only for acute but also for chronic viral infections. In the case of chronic infection entry inhibition prohibits infection from spreading to naive cells, which together with antiviral therapy eliminates infected cells providing higher chance of cure. For example, in HIV infection this has been accomplished with the interference of virus entry using a gp41 protein derived peptide, enfuvirtide, which prevents fusion of the virus with the host cellular membrane [47]. Virus entry inhibition also provides an opportunity to prevent recurrent hepatitis B after liver transplantation. Previous studies demonstrate that acylated HBV preS-derived lipopeptides targeting viral envelope protein components could prevent the interaction of HBV with its cellular receptor, thus preventing de novo HBV infection in humanized mice [48]. Since hepatitis Delta virus uses the same receptor as HBV, HBV entry inhibitors are equally effective against both viruses [49]. Although there is no evidence indicating that HBV can propagate by cell-to-cell transmission, numerous enveloped viruses have been shown to employ modes of spreading involving both direct cell-cell transmission and the release of progeny viruses into the extracellular space [50]. Further study showed the myristoylated preS-derived peptide Myrcludex-B could block HBV cell-to-cell dissemination among human hepatocytes in the liver of humanized mice [51]. Similarly, in the context of HCV infection, an entry inhibitor targeting viral receptor claudin-1 used in monotherapy was shown to cure chronic infection in the infected humanized mice [52].

Altogether, recent studies results indicate that inhibition of HBV/HCV entry or binding, in combination with established therapies, might have potential applications in preventing vertical transmission during birth, reinfection after liver transplantation, or chronic HBV infection. In both HBV and HCV infection, hepatocyte turnover likely results in the reduced infection. Even more importantly, a recent clinical trial using entry inhibitor Myrcludex-B to treat patients with chronic hepatitis Delta has demonstrated that an entry inhibitor alone or in combination with IFN-*α* has a pronounced antiviral effect [53]. By blocking reinfection and protecting uninfected hepatocytes from de novo infection, IFN-induced viral entry inhibitors complement already known antiviral effects of IFN-*α* and their identification may open perspectives for novel therapeutic approaches for HBV and HCV infection.

## 5. Conclusion

In conclusion, this study reveals that IFN-*α* is able to induce soluble factors that bind to heparan glycosaminoglycans and lead to the inhibition of HBV and HCV binding and thus unravel a novel antiviral mechanism of action of interferons.

## Figures and Tables

**Table 1:** Primers for qPCR.

Name	Sequence 5′-3′
cccDNA 92 fw	GCCTATTGATTGGAAAGTATGT
cccDNA 2251 rev	AGCTGAGGCGGTATCTA
PRNP fw	GACCAATTTATGCCTACAGC
PRNP rev	TTTATGCCTACAGCCTCCTA
rcDNA1745 fw	GGAGGGATACATAGAGGTTCCTTGA
rcDNA1844 rev	GTTGCCCGTTTGTCCTCTAATTC
